# Mechanisms and Risk Factors Contributing to Visual Field Deficits following Stereotactic Laser Amygdalohippocampotomy

**DOI:** 10.1159/000502701

**Published:** 2019-10-16

**Authors:** Natalie L. Voets, Ivan Alvarez, Deqiang Qiu, Christopher Leatherday, Jon T. Willie, Stamatios Sotiropoulos, Ezequiel Gleichgerrcht, Leonardo Bonilha, Nigel P. Pedersen, Nadja Kadom, Amit M. Saindane, Robert E. Gross, Daniel L. Drane

**Affiliations:** _a_Wellcome Centre for Integrative Neuroimaging, FMRIB Centre, Nuffield Department of Clinical Neurosciences, University of Oxford, Oxford, United Kingdom; _b_Department of Radiology and Imaging Sciences, Emory University School of Medicine, Atlanta, Georgia, USA; _c_Sir Peter Mansfield Imaging Centre, School of Medicine, University of Nottingham, Nottingham, United Kingdom; _d_Department of Neurology, Medical University of South Carolina, Charleston, South Carolina, USA; _e_Department of Neurology, Emory University School of Medicine, Atlanta, Georgia, USA; _f_Children's Hospital of Atlanta, Atlanta, Georgia, USA; _g_Department of Neurosurgery, Emory University School of Medicine, Atlanta, Georgia, USA; _h_Department of Pediatrics, Emory University School of Medicine, Atlanta, Georgia, USA; _i_Department of Neurology, University of Washington School of Medicine, Seattle, Washington, USA

**Keywords:** Temporal lobe epilepsy, Laser ablation, Laser interstitial thermal therapy, Diffusion tensor imaging, Interstitial ablation, Optic radiations

## Abstract

Selective laser amygdalohippocampotomy (SLAH) is a minimally invasive surgical treatment for medial temporal lobe epilepsy. Visual field deficits (VFDs) are a significant potential complication. The objective of this study was to determine the relationship between VFDs and potential mechanisms of injury to the optic radiations and lateral geniculate nucleus. We performed a retrospective cross-sectional analysis of 3 patients (5.2%) who developed persistent VFDs after SLAH within our larger series (*n* = 58), 15 healthy individuals and 10 SLAH patients without visual complications. Diffusion tractography was used to evaluate laser catheter penetration of the optic radiations. Using a complementary approach, we evaluated evidence for focal microstructural tissue damage within the optic radiations and lateral geniculate nucleus. Overablation and potential heat radiation were assessed by quantifying ablation and choroidal fissure CSF volumes as well as energy deposited during SLAH. SLAH treatment parameters did not distinguish VFD patients. Atypically high overlap between the laser catheter and optic radiations was found in 1/3 VFD patients and was accompanied by focal reductions in fractional anisotropy where the catheter entered the lateral occipital white matter. Surprisingly, lateral geniculate tissue diffusivity was abnormal following, but also preceding, SLAH in patients who subsequently developed a VFD (all *p* = 0.005). In our series, vision-related complications following SLAH, which appear to occur less frequently than following open temporal lobe ­surgery, were not directly explained by SLAH treatment parameters. Instead, our data suggest that variations in lateral geniculate structure may influence susceptibility to indirect heat injury from transoccipital SLAH.

## Introduction

Selective laser amygdalohippocampotomy (SLAH) is a recently developed minimally invasive surgical approach for the treatment of drug-resistant temporal lobe epilepsy (TLE). SLAH aims to achieve ablation of epileptogenic structures within the medial temporal lobe (MTL) while reducing secondary damage to overlying cortical and white matter structures incurred during traditional “open” resection [1]. Emerging data indicate lower rates of decline in naming, object/face recognition and verbal memory following SLAH compared to open resections [2, 3, 4, 5, 6] (reviewed in [7]).

Visual field deficits (VFDs) are a common complication of TLE surgery. After open resections, including anterior temporal lobectomy and selective amygdalohippocampectomy, measurable VFDs occur in approximately 75% of patients [8]. Incidence rates vary from 48–83% after classical anterior temporal lobectomy to 49–53% following selective approaches [8, 9]. Postoperative inflammation and edema may contribute in the first few weeks following surgery [10]. However, persistent VFDs following open resections have historically been attributed to direct surgical damage to the anteriormost fibers of the optic radiations (“Meyer's loop”) [11]. Such damage commonly leads to a contralateral superior, frequently homonymous, subtotal-to-total quadrantanopia. Estimates of the anatomical course of Meyer's loop, based on diffusion-weighted MRI tractography, have shown high accuracy in predicting surgical risks to the optic radiations in individual patients [12]. Intraoperative visualization of tractography-derived optic radiation fibers, furthermore, substantially reduces the incidence of VFDs following open surgery [13].

In contrast, during SLAH an optical fiber is inserted into the hippocampus and amygdala by means of a narrow (1.6 mm) diameter catheter, commonly via a transoccipital approach [14, 15]. Consequently, no surgical access corridor is created through inferolateral temporal tissue, avoiding resection or retraction of ventral optic radiation fibers in the anterior temporal lobe. In several early series, approximately 11% of patients nonetheless experience VFDs following SLAH (range: 4.8–20%) [1, 14, 15, 16, 17, 18, 19].

Multiple mechanisms for VFDs have been proposed. It has been speculated that injury to the lateral geniculate nucleus (LGN) might arise directly through catheter misplacement [14, 17], or indirectly by thermal injury [17] or cytotoxic edema [17, 19] associated with the laser catheter route and power utilized. A second proposed mechanism is transection or ablation of the posterior optic radiations by the laser catheter [18]. Evidence that SLAH induces long-lasting disruption of optic radiation white matter microstructure, however, is currently lacking, while damage sufficient to affect vision is thought unlikely based on the small diameter of the catheter [20]. Finally, VFDs may reflect a combination of direct and thermal injury, including anterior optic radiation fibers overlying the posterolateral aspect of the amygdala or lateral to the inferior temporal horn.

Here, we investigated these hypothesized mechanisms in 3 among 58 consecutive patients who developed de novo visual symptoms and had >12 months of follow-up after SLAH. We acquired longitudinal pre-, intra- and postoperative imaging data in VFD patients for comparison against both healthy controls and SLAH patients who did not develop visual symptoms. Our aims were to determine whether patients who experienced VFDs after SLAH share a common mechanism of injury enabling procedural adaptations to minimize VFDs following SLAH.

## Materials and Methods

### Participants

We retrospectively reviewed a cohort of 58 patients who underwent SLAH at Emory University Hospital between July 1, 2011, and June 30, 2016, to identify patients reporting new visual symptoms following treatment. We have recently described the 1-year surgical outcomes of this cohort [1], among whom 5 visual complications were recorded. In 1 patient, symptoms were transient and visual fields normal on confrontational testing at the clinical follow-up. A second patient reported inconsistent subjective visual complaints, but no objective deficit was found, and complaints resolved by the 1-year follow-up. The remaining 3 patients (5.2%, cases A–C) were recorded as having incurred an objective and persistent VFD.

Case A had undergone a previous procedure which resulted in ablation of the right parahippocampal gyrus (but not the hippocampus), prior to taking part in this study [1, 14]. When this failed to provide seizure control, a second procedure was performed to ablate the right hippocampus, which resulted in an unexpected near complete persistent left homonymous hemianopsia. This was the first patient in the study, and no preablation imaging data were collected. Cases B and C experienced a right superior quadrantanopsia after undergoing singular left SLAH procedures. In both cases, initial visual symptoms improved but continued over the 1-year postoperative interval. The degree of VFD was confirmed through Humphrey visual field assessments in cases A and B (online suppl. Fig. S1; see www.karger.com/doi/10.1159/000502701 for all online suppl. material). Case C did not undergo a formal neuro-ophthalmological examination.

A group of 15 healthy controls (HCs, mean age 35.7 years, range 18–54, 2 men) was included for normative values. Inclusion criteria were: aged 18 years and over, speaking English as their first language. HCs were excluded if they had a history of substance abuse or any neurological or psychiatric illness.

A second “surgical control” (SC) group was included to establish typical variations in optic radiation parameters before and following SLAH. The SC patients had undergone the same SLAH procedure for medial TLE but did not self-report visual deterioration. Out of the larger group of SLAH patients, all those who had matched pre- and postoperative diffusion MRI data, acquired on the same scanner as the VFD patients, were selected for the SC group. Ten patients met these criteria (5 men, 5 women, mean age 42.2 years, range 18–67 years).

All patients underwent a comprehensive epilepsy evaluation by a multidisciplinary clinical team, which included MRI, extended video telemetry, inpatient video-EEG, 18-fluorodeoxyglucose positron emission tomography, neuropsychological testing, functional MRI for language and memory evaluation, and, where indicated, Wada testing and invasive electroencephalographic recordings. Patients had either radiological evidence of unilateral hippocampal atrophy or normal clinical MRI with MTL hypometabolism. All patients had scalp and/or invasive electroencephalographic evidence of seizures implicating the ipsilateral MTL. Patients who had prior open resections, additional confounding pathology (e.g., head trauma, encephalitis) or MRI abnormalities other than mesial temporal sclerosis were excluded from the SC group. Participant data are presented in Table 1.

### Surgical Procedure

The SLAH procedure was performed under general anesthesia using the Visualase^TM^ system (Medtronic, Lewiston, CO, USA) as described previously [14]. In brief, a 1.6-mm laser cooling catheter was implanted using either an MRI-guided trajectory frame or a standard stereotactic head frame. An intraoperative volumetric T1-weighted scan was acquired to confirm the accurate positioning of the catheter along the planned trajectory from the lateral occipital cortex through the length of the hippocampus at the level of the hippocampal body. Using real-time thermal maps generated on the Visualase^TM^ workstation, a 15-W 980-nm wavelength diode laser was used to perform a first ablation anteriorly. Additional ablative pulses were then delivered at 8- to 10-mm intervals by retracting the optic fiber posteriorly along the length of the hippocampus until a complete ablation was evidenced on diffusion-weighted, FLAIR, and postgadolinium contrast T1-weighted sequences. For additional details, see the online supplementary Materials.

### MRI Acquisition

Baseline and 1-year follow-up MRI data were acquired on a 3-T Siemens Trio MRI. Diffusion MRI data were acquired along 64 noncolinear directions at a b-value of 1,000 s/mm^2^ and a 2 × 2 × 2 mm voxel size. A high-resolution T1-weighted anatomical scan was acquired for coregistration of the diffusion data. For MRI acquisition parameters and preprocessing steps, see the online supplementary Materials.

### Analyses

We performed 5 analyses, exploring previously proposed evidence for transection of the optic radiations [18], thermal injury to the optic radiations [16, 17], or injury to the LGN. Further technical details for each step are reported in the online supplementary Materials.

#### Optic Radiation Injury Load

**Fig. 1:**
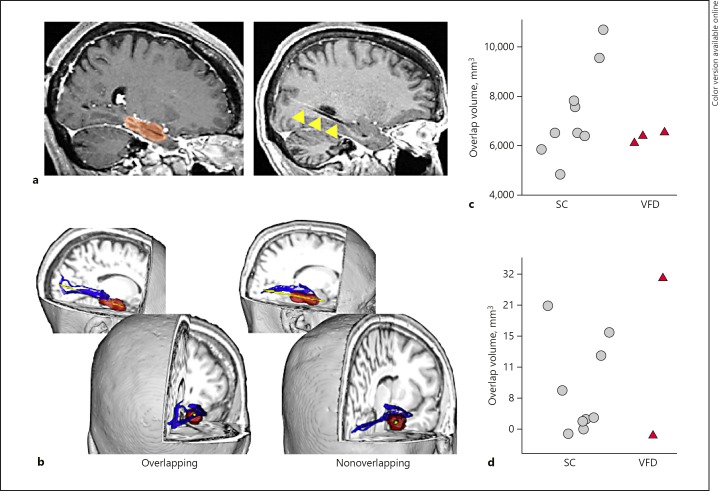
Ablation volumes and optic radiation injury load following SLAH. **a** Example delineation of the ablation zone on the intraoperative postgadolinium contrast scan (red mask) and laser catheter trajectory (yellow arrows). **b** Representative patients with and without spatial overlap between the laser catheter trajectory (yellow) and the optic radiations (blue). The ablated zone in each patient is shown in red. Views are shown from the side (top) and from behind (below) to appreciate the trajectory of the catheter relative to the optic radiations. **c** Total ablation volume was not larger in patients who developed a visual field deficit (VFD, *n* = 3) than in surgical control patients (SC, *n* = 9). The volume of intersection between the laser catheter trajectory and the preoperative optic radiations was substantially greater in 1 VFD patient (case C) than in SC patients (**d**).

To estimate the optic radiation “injury load,” we reconstructed the optic radiations in each hemisphere in each participant through probabilistic tractography using the FMRIB Software Library (FSL). Next, the trajectory of the laser catheter was manually traced on the intraoperatively acquired structural image (Fig. 1a) available for 9 of 10 SC patients and both VFD patients with pre- and intraoperative data. For each patient, the laser catheter mask was registered to their preoperative structural image to calculate the volume (mm^3^) and percentage of overlap between the catheter and the pre-SLAH tractography-generated optic radiations. To exclude the possibility that larger surgical volumes, encompassing the optic radiations, accounted for the occurrence of VFDs, the ablation zone volume was also manually delineated on the periablation postgadolinium contrast T1-weighted scan. The ablation zone was defined as all voxels showing postcontrast enhancement, including all of the enhancing ring but excluding the choroid plexus when possible (Fig. 1a).

#### Tract-Based Spatial Statistics

**Fig. 2:**
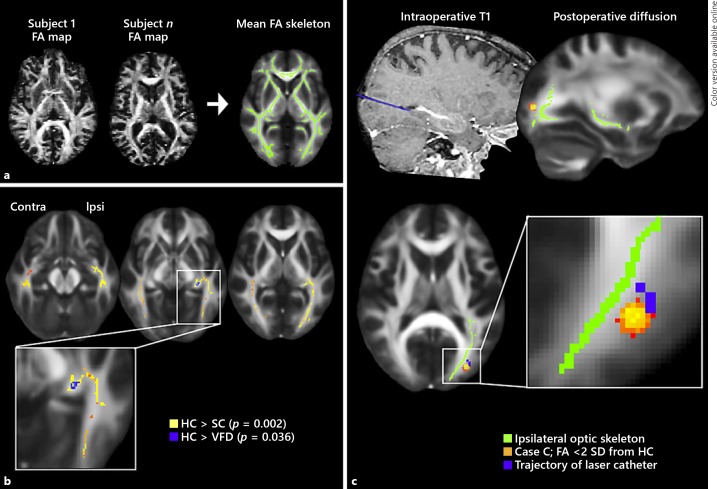
Optic tract-based voxel-wise fractional anisotropy (FA) in patients with and without visual field deficits (VFDs). **a** For tract-based spatial statistics analysis, every participant's FA map was aligned to a standard template, from which a core white matter “skeleton” was created. **b** Voxel-wise analysis along the optic radiations identified reduced FA in all patients following selective laser amygdalahippocampotomy, both with (*n* = 3) and without (*n* = 10) visual field deficits, when compared to healthy controls (HCs, *n* = 15) (all *p* < 0.05). **c** One patient (case C) who developed a visual field defect showed focal FA reductions immediately adjacent to the laser catheter entry point. Other optic radiation voxels that overlapped with the laser catheter trajectory did not show reduced FA.

To test for focal disruptions within the optic radiations, we quantified fractional anisotropy (FA), indexing white matter tissue microstructure [21], using voxel-wise statistical analyses implemented in tract-based spatial statistics. Between-group comparisons of FA were performed using the conventional tract-based spatial statistics processing pipeline and constrained to the optic radiations using masks from the Jülich histological atlas (Fig. 2). In order to examine FA changes ipsilateral and contralateral to the side of seizure onset/SLAH, the hemispheres of patients with right TLE were flipped to standardize the treatment hemisphere to the left across all patients.

#### Choroidal Fissure CSF Volumes

**Fig. 3:**
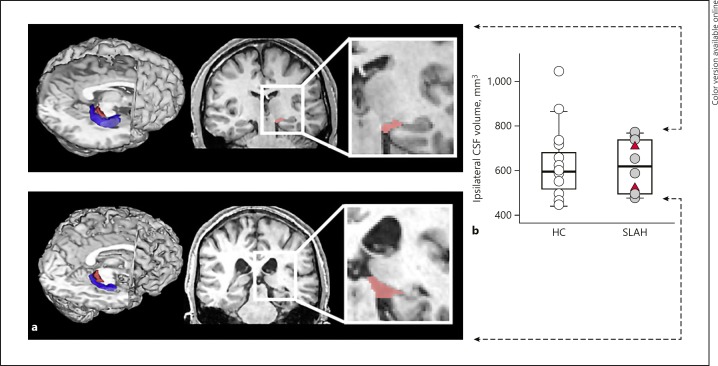
Presurgical choroidal fissure cerebrospinal fluid (CSF) volumes. **a** CSF volumes in the choroidal fissure prior to selective laser amygdalohippocampotomy in a patient with low (top row) and a patient with high (bottom row) ipsilateral choroidal fissure CSF volume. **b** Both patients (gray dots, SLAH) and healthy controls (white dots, HCs) exhibited a wide range of CSF volumes. However, both patients who developed a visual field deficit following SLAH (red triangles) showed CSF volumes within the normal range.

To quantify choroidal fissure CSF volume, we created a region of interest on the template MNI brain, using anatomical landmarks to define the temporal part of the choroidal fissure (Fig. 3). The number of voxels within this region of interest on each individual's structural volume was converted into CSF volume (mm^3^), adjusted for intracranial volume.

#### LGN Tissue Microstructure Measurements

**Fig. 4:**
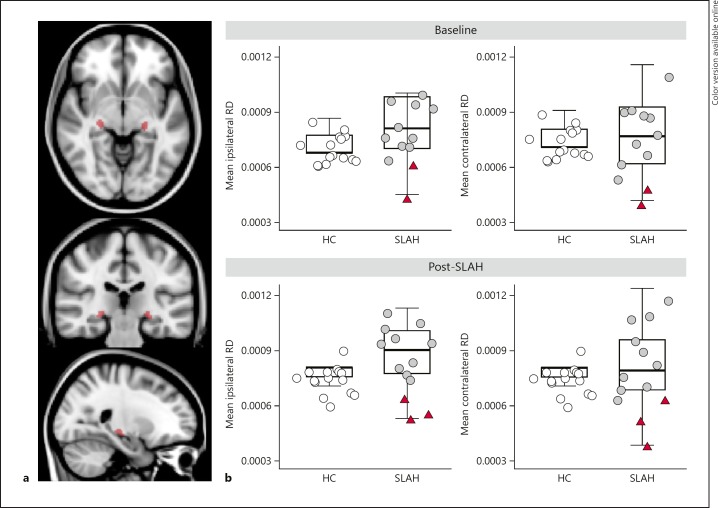
Diffusion microstructure parameters of the lateral geniculate nucleus. **a** Lateral geniculate nucleus (LGN) region of interest masks (red) from the Jülich histological atlas. **b** Diffusion parameters (fractional anisotropy, radial diffusivity RD, axial diffusivity) were from the LGN in healthy controls (HCs) and patients undergoing selective laser amygdalohippocampotomy (SLAH). SLAH patients who experienced a visual field deficit (VFD) are plotted in red triangles alongside patients who did not experience a VFD (gray circles). Reduced RD differentiated VFD patients from surgical controls without visual symptoms (ipsilateral LGN *F*(1) = 23.47, *p* = 0.001) and were already present prior to SLAH.

Potential indirect injury to the LGN was assessed by measuring microstructural diffusion parameters within the LGN. We used the Jülich probabilistic histology atlas to define objective LGN masks for each hemisphere separately (Fig. 4) and extract, in each individual, the average FA, radial diffusivity (RD, thought to reflect myelination) and axial diffusivity (AD, a marker of axonal integrity and/or gliosis) [22]. Values were averaged across the two hemispheres in HCs to enable comparisons of changes in the LGN ipsilateral versus contralateral to SLAH in patients.

#### SLAH Parameters

The SLAH ablation parameters were available for 2 of 3 VFD patients and 8 of 10 SC patients. For each available data set, we calculated the energy (joules) deposited to the treated locations by multiplying the power (watts) and the duration (seconds) of each ablation (online suppl. Materials).

### Statistical Analyses

Statistical analyses were performed using SPSS v25 (IBM®). Shapiro-Wilk tests revealed some diffusion and volume measurements did not meet assumptions of normality, therefore, data were Box-Cox transformed prior to group-level analyses. Age, total ablation volumes, optic radiation injury parameters and choroidal fissure CSF volumes were compared using independent samples*t* tests. Multivariate analyses of covariance, covarying for age, were used to compare LGN diffusion variables and SLAH laser settings. Multivariate test results are reported before and after Bonferroni correction for multiple comparisons. *p* values ≤0.05 were considered statistically significant.

Voxel-wise analyses of diffusion parameters within tract-based spatial statistics were performed through nonparametric permutation testing using the FSL tool “randomize.” Between-group comparisons (5,000 permutations) were performed using unpaired *t* tests. Threshold-free cluster enhancement was used to identify clusters on the skeleton with *p* values <0.05 (family-wise error corrected for multiple comparisons).

To assess abnormalities in each individual VFD patient, we determined whether the values in individual VFD patients were greater than 2 standard deviations (SD) from the mean observed in each control group (HC/SC).

## Results

Age did not differ between the 3 VFD patients and the control groups (HC: *t*(df 16) = −1.9, *p* = 0.07; SC: *t*(11) = −0.78, *p* = 0.45). Ablation volumes did not differ between SC (mean: 7,204 ± 1,822 mm^3^) and VFD patients either (mean: 6,319 ± 164 mm^3^) (*t*(10) = 1.40, *p* = 0.19) (Table 1, Fig. 1c).

### Optic Radiation Injury Load

The volume of optic radiations intersected by laser catheter did not differ between the 2 VFD patients with preoperative diffusion data (mean: 16 ± 22.6 mm^3^) and the SC group (mean: 6.1 ± 8.0 mm^3^, *t*(9) = −1.18, *p* = 0.27). There was no difference in the percentage of optic radiation fibers affected by the catheter trajectory either (<1% in both groups, *t*(9) = 1.04, *p* = 0.33) (online suppl. Table S1). At the individual level, however, the optic radiation lesion load in case C (32 mm^3^) was abnormally high relative to the SC group (Fig. 1d). For completeness, we ruled out overlap between the ablation zone and the probabilistic tractography-reconstructed optic radiations as a potential contributor to VFDs. No overlap was found (mean overlap in VFD patients: 0 mm, mean overlap in SC: 2.4 mm, range 0–20 mm).

### Tract-Based Spatial Statistics

At the group level, SC patients showed bilaterally reduced FA relative to HC throughout the optic radiations following SLAH (ipsilateral permutation *p* value = 0.002, contralateral *p* = 0.004, family-wise error corrected for multiple comparisons) (Fig. 2b). The VFD patients showed focally reduced ipsilateral FA compared to HCs (*p* = 0.036) but did not differ when compared to SLAH patients without VFDs. When considered individually, 1 VFD patient (case C) had abnormally low FA values on the operated side compared to HC and SC ranges (online suppl. Table S1). The abnormal values localized to the lateral occipital white matter directly adjacent to the trajectory along which the catheter was inserted (Fig. 2c). Examination of the presurgical diffusion data confirmed that this focal disruption emerged only after SLAH.

### CSF Volumes

Baseline (pre-SLAH) choroidal fissure CSF volumes in the ablated hemisphere were not significantly different between groups (mean SC: 613.5 ± 126.9 vs. mean VFD: 606.8 ± 127, *t*(10) = 0.069, *p* = 0.95) (online suppl. Table S2). At the individual level, the choroidal fissure CSF volumes for both VFD patients with available baseline data remained within normal ranges (Fig. 3).

### LGN Diffusion Parameters

To test for LGN thermal injury, we evaluated diffusion parameters (FA, RD, AD) within the LGN as markers of tissue microstructure. Post-SLAH diffusion parameters differed between groups both in the ipsilateral LGN (*F*(6, 46) = 17.03, *p* < 0.001) and the contralateral LGN (*F*(6, 46) = 2.83, *p* = 0.020) (Table 2). Post hoc analyses revealed reduced ipsilateral RD values in the 3 VFD patients following SLAH when compared to both the HC (*F*(1) = 18.8, *p* = 0.001) and SC groups (*F*(1) = 23.47, *p* = 0.001) (Fig. 4). Bilateral RD differences remained significant after Bonferroni correction for comparison of 3 diffusion parameters (all *p* < 0.003).

Because abnormalities in diffusion parameters were observed bilaterally, and are therefore difficult to ascribe to unilateral SLAH, we investigated whether LGN diffusion parameters in the available VFD patients (*n* = 2) differed already at baseline (before ablation). Indeed, preoperative LGN diffusion parameters showed a significant effect of group (ipsilateral: *F*(6, 44) = 8.57, *p* < 0.001; contralateral *F*(6, 44) = 5.68, *p* < 0.001). Post hoc analyses showed that patients who would subsequently go on to develop a VFD following SLAH had lower RD values prior to treatment than patients who did not (ipsilateral, *F*(1) = 14.11, *p* = 0.005); contralateral, (*F*(1) = 17.96, *p* = 0.001); the SC group did not differ from HC. All baseline findings remain significant after Bonferroni correction for 3 comparisons (all *p* < 0.015).

At the single subject level, all VFD patients had abnormally low RD values ipsilateral to SLAH compared to the normal ranges (Table 2). These RD abnormalities were also present at baseline in both cases with available preoperative diffusion data (cases B and C) and did not change between the pre- and postoperative time points in these two cases.

### SLAH Parameters

Finally, the amount of SLAH energy deposited as a whole or within MTL subregions did not differ between the VFD patients (*n* = 2) and SC patients (*n* = 8) with available data (*F*(4, 5) = 1.74, *p* = 0.28) (online suppl. Table S2). The SLAH settings for individual VFD patients were among the lowest of all patients, contradicting the hypothesis of greater energy deposition leading to VFD (online suppl. Fig. S3).

## Discussion

Cause(s) under debate as an explanation for VFDs following SLAH include: (1) direct transection of optic radiations, (2) thermal ablation of optic radiations, and (3) thermal injury to the LGN related to low absorptive CSF concentrations or high levels of heat energy deposited. In our series, neither pretreatment choroidal fissure CSF volume nor energy delivered to the MTL distinguished patients who developed a VFD following SLAH from patients who did not. Instead, 1 patient had evidence of catheter-related damage to the optic radiations. Moreover, differences in LGN diffusivity distinguished all 3 VFD patients from surgical controls and were evident prior to treatment. Although we did not uncover one consistent causative mechanism, our findings indicate that VFDs are not directly explained by SLAH treatment parameters; VFDs may have multiple causes and potentially reflect pre-existing susceptibility factors.

VFDs are the most frequently cited complication of surgical treatments for epilepsy, including SLAH [1, 14, 16, 17, 18, 19]. Based on the typical posterolateral catheter insertion trajectory, SLAH poses a theoretical risk of damaging the posterior optic radiations [18]. Consistent with this notion, we observed overlap, albeit only 2%, between the laser catheter and optic radiations in 1 of our 3 patients who developed a VFD. In this patient, post-treatment white matter disruption, indexed through FA, colocalized with the catheter's entry point into the lateral occipital lobe white matter. This patient experienced a right superior quandrantanopsia, consistent with injury to the posterior optic radiations. In contrast, several SLAH patients showed evidence of optic radiation transection without developing symptomatic VFDs. Another patient developed a homonymous hemianopsia without detectable optic radiation injury. Consequently, persisting VFDs can occur without accompanying evidence for long-term optic radiation disruption after SLAH.

Misplacement of the laser catheter has been advanced as a potential alternative mechanism for visual complications [16]. In 1 patient (case A), the initial trajectory of the catheter was suspected to have penetrated the LGN. The precise location of the LGN is challenging to identify on standard (T1/T2-weighted) structural scans. Instead, visualizing the catheter trajectory in this patient relative to a probabilistic atlas highlighted potential ablation at the inferolateral border of the LGN (online suppl. Fig. S2). Since we did not have preablation imaging for this patient, we cannot determine whether the patient's postoperatively abnormal LGN diffusion parameters reflect a *change* indicative of direct injury. However, the patient's postablation LGN values were similar to the other 2 VFD patients, and no abnormalities were observed in this patient's optic radiation FA. Consequently, no clear-cut cause for their homonymous hemianopsia was identified.

Alternatively to *direct* injury, the proximity of the LGN to the hippocampus makes it susceptible to *indirect* injury [14, 17] through overablation, heat radiation [16] and/or cytotoxic edema [23]. However, total ablation volume did not differ between our VFD patients and our asymptomatic SLAH group. A previous study also concluded that VFD in their case report was not likely the result of a larger ablation [17]. Instead, the authors proposed that smaller choroidal fissure CSF volumes could reduce the protective effect (“heat sink”) of CSF around the ablated hippocampus. We did not identify smaller pre-SLAH choroidal fissure CSF spaces among patients who developed VFDs compared to patients who did not.

In contrast, a measure of LGN tissue microstructure − RD − was abnormal bilaterally and predating SLAH in patients who developed VFDs. There is a 2- to 3-fold intraindividual difference in the size of the optic tract, LGN, optic radiation and area of the recipient primary visual cortex [24]. This variation raises the possibility that diffusivity differences in VFD patients may in part reflect a small LGN. Given the lack of LGN contrast in T1-weighted scans, our atlas-based LGN mask − when applied to data of patients with small LGNs − may have included more white matter adjacent to the LGN or resulted in partial sampling of the adjacent pulvinar nucleus, which is pathologically damaged in some patients with chronic TLE [25]. Alternatively, an intriguing though speculative proposal is that preoperative differences in LGN structure may heighten susceptibility to VFDs, for example by amplifying the effects of heat radiation within the LGN (which, however, did not show diffusivity change pre-to-post SLAH in our atlas-derived masks). To verify this hypothesis, imaging sequences (such as proton density) optimized to the LGN are needed. Further exploration of the potential relationship between LGN and CSF volumes, tissue heat dissipation and behavioral complications across a larger group of affected patients merits further examination.

Limitations of our study include the small sample size, reflecting the low incidence of VFDs in our series (3/58). Secondly, it is not standard practice to measure visual fields prior to treatment or in asymptomatic patients, which might have identified pre-existing visual disturbances [26] exacerbated by SLAH. Finally, imaging patients 1 year following SLAH minimizes overestimation of VFDs resulting from transient inflammatory responses [27], but, conversely, we cannot exclude recovery of white matter parameters in the intervening year. Indeed, while case A's homonymous hemianopsia remained unchanged, case B's quadrantanopsia improved and case C's quadrantanopsia was constricting, though both persisted at 1 year. Serial longitudinal scans will be important to correlate white matter measures with potential symptom improvements in VFDs over time.

## Conclusion

We performed to our knowledge the first comparison of mechanisms potentially associated with VFDs following SLAH. Diffusion imaging data support the possibility that direct injury to the optic radiations during catheter placement contributes to VFDs in some patients. This may warrant consideration of tractography during preoperative planning, and the selection of trajectory may be a modifiable risk factor. In our cumulative experience, a hippocampal ablation trajectory that penetrates the hippocampal body from an inferior-to-superior path avoids the ventricle and decreases the proximity of the fiber to the LGN, providing an additional margin of safety. However, additional mechanisms likely impact on visual outcomes after SLAH, since no measurable cause was found for the homonymous hemianopsia in 1 patient in whom the catheter *may* have abraded the LGN. Importantly, SLAH treatment parameters did not account for visual complications in our series. Instead, we propose that natural variability in the size of the LGN and related structures may anticipate visual complications following SLAH, potentially by lowering the threshold for heat injury either independently of, or cumulative with, other factors such as small choroidal fissure CSF volumes.

## Statement of Ethics

The Emory University Institutional Review Board approved this study. All participants gave informed written consent to take part.

## Disclosure Statement

D.L.D. manages the Neuroimaging and Neuropsychology Core Labs of an FDA multisite trial of laser ablation sponsored by Medtronic Inc., the manufacturer of the Visualase system. He also received a research grant from them in the past. J.T.W. and R.E.G. serve as consultants to Medtronic Inc. and receive compensation for these services. Medtronic Inc. develops products related to the research described in this paper. The terms of this arrangement have been reviewed and approved by Emory University in accordance with its conflicts of interest policies. Medtronic had no role in the collection, analysis or interpretation of these study data.

## Funding Sources

This work was supported by grants received by Dr. Drane from the National Institutes of Health (R01NS088748, K02NS070960, L30 NS080215) and Medtronic Inc. (A1225797BFN), a research grant from Visualase Inc. (Agency Award No.: VIS-10-001) and a Shared Instrumentation Grant (S10: Grant 1@10OD016413-01) to the Emory University Center for Systems Imaging Core. N.L.V. gratefully acknowledges personal support from the National Institutes of Health Research Oxford Biomedical Research Centre and the Wellcome Centre for Integrative Neuroimaging, which is supported by core funding from the Wellcome Trust (203139/Z/16/Z). I.A. was supported by the UK Medical Research Council (MR/K014382/1). N.P.P. was supported by the National Institutes of Health (K08NS105929).

## Author Contributions

N.L.V., I.A., L.B. and D.D. conceived and designed the analysis. D.D., D.Q., C.L., A.S. and N.K. acquired the data. S.S., J.W. and R.E.G. contributed data or analysis tools. N.L.V. analyzed the data with input from I.A., L.B., N.P.P., J.W. and R.E.B. N.L.V. drafted the paper. All authors contributed to interpreting the findings and editing the manuscript.

## Supplementary Material

Supplementary data

## Figures and Tables

**Table 1:** Clinical and demographic data

	Age, years	Gender	Age at onset, years	Duration of epilepsy, years	Clinical MRI	Side of seizure onset/SLAH	Ablation volume, mm^3^
*Patient cases*							
Case A	44	F	36	8	Right hippocampal signal change (no atrophy)	R	6,302
Case B	43	M	40	3	Left MTS	L	6,490
Case C	65	F	5	60	Left MTS	L	6,164

*Surgical controls*							
*n* = 10	42.2±17.3	4 M, 6 F	12.3 ± 9.6	20±19.4	8 MTS, 1 signal change (no atrophy), 1 normal MRI	3 R, 7 L	7,203±1,823

*Healthy controls*							
*n* = 15	35.7±12.4	2 M, 13 F	−	−	Normal	-	-

MRI, magnetic resonance imaging; SLAH, selective laser amygdalohippocampotomy; MTS, medial temporal sclerosis.

**Table 2:** Lateral geniculate nucleus diffusion parameters

	After ablation				Before ablation			
	ipsi		contra		ipsi		contra	
	RD	AD	RD	AD	RD	AD	RD	AD
HC	0.0008±0.00007	0.0013±0.00009	0.0008±0.00007	0.0013±0.00009	0.0008±0.0007	0.0013±0.00009	0.0008±0.0007	0.0013±0.00009
SC	0.0009±0.00012	0.0014±0.00011	0.0009±0.00019	0.0015±0.00016	0.0009±0.00013	0.0014±0.00013	0.0008±0.00017	0.0014±0.00014
Case A	0.00066*	0.0014	0.00054	0.00126	-	-	-	-
Case B	0.0005*	0.0013	0.00038*	0.0014	0.00046*	0.00128	0.00041*	0.00137
Case C	0.0006*	0.0019	0.00068	0.00043	0.0006*	0.0014	0.00048	0.0015

Lateral geniculate nucleus (LGN) diffusion MRI parameters in healthy controls (HC, *n =* 15), surgical controls (SC, *n =* 10) and patients who developed visual symptoms following ablation (*n =* 3). Mean values and standard deviations of radial diffusivity (RD) and axial diffusivity (AD) were sampled from histologically defined (Jülich atlas) masks of the LGN. Individual visual field defect patient values greater than 2 SD from the control groups are indicated in bold font with an asterisk.
