# Supplementary material for: Mechanisms and Risk Factors Contributing to Visual Field Deficits following Stereotactic Laser Amygdalohippocampotomy
Source: Stereotact Funct Neurosurg. 2019 Oct 16;97(4):255–65. doi: 10.1159/000502701 (PMC6979425; doi:10.1159/000502701)
Supplement: Supplementary file 1 — Supplementary data [file sfn-0097-0255-s01.docx]

Supplementary materials for Voets et al., “*Mechanisms and risk factors contributing to visual field deficits following stereotactic laser amygdalohippocampotomy”.*

**Supplementary Methods:**

*Surgical procedure*

The SLAH procedure was performed under general anaesthesia using the Visualase^TM^ system (Medtronic, Lewiston, CO) as described previously [1]. A 1.6mm laser cooling catheter was implanted using either a MRI-guided trajectory frame or a standard stereotactic head frame. For the stereotactic frame cases, intraoperative C-arm fluorography was performed with the insertion rod in place to confirm accuracy and the trajectory adjusted as needed followed by laser catheter and optical fiber insertion. An intraoperative volumetric T1-weighted scan was acquired to confirm the accurate positioning of the catheter along the planned trajectory from the lateral occipital cortex through the length of the hippocampus at the level of the hippocampal body and descending into the amygdala. Patients implanted in the MR scanner with the MRI-guided trajectory frame underwent T1 imaging first with a ceramic stylet in place to confirm accuracy and the trajectory was then adjusted if needed. In all cases, the 0.73mm optical fiber with 10mm long diffuser tip was used. Using real-time thermal maps generated on the Visualase^TM^ workstation, a 15W 980nm wavelength diode laser was used to perform a first ablation anteriorly. Additional ablative pulses were then delivered at 8 – 10mm intervals by retracting the optic fiber posteriorly along the length of the hippocampus until a complete ablation was evidenced on diffusion-weighted, FLAIR, and post-gadolinium contrast T1-weighted sequences.

*Diffusion data acquisition and pre-processing*

Diffusion MRI data were acquired using an echo planar imaging sequence (TE = 93ms, TR = 8700ms, 2 × 2 × 2mm voxel size, GRAPPA acceleration factor = 2) with a single shell sequence (*b* = 1000 s/mm^2^, 64 directions, 6 *b* = 0 volumes). The diffusion data were acquired twice, in phase-reversed encoding directions (right-to-left and left-to-right), for *post-hoc* image distortion correction [2]. The diffusion scan time was 09:52 minutes per acquisition (i.e. 19 minutes in total). Acquisition parameters for the T1-weighted MPRAGE anatomical scan were: TR = 1970ms, TE = 3.06ms, 0.9 × 0.9 × 1mm voxel size, FOV = 240mm.

Diffusion data were pre-processed using standard FMRIB Software Library tools (FSL, v5.0) (<https://fsl.fmrib.ox.ac.uk/fsl/>). Following visual quality inspection, the susceptibility-induced field was estimated from the non-diffusion-weighted phase-reversed diffusion image pairs using the tool ‘topup’ [2]. Using the estimated fieldmap, the diffusion-weighted data were corrected for susceptibility-induced distortions, eddy currents and head motion using the tool ‘eddy’ [3]. The two diffusion datasets were averaged to maximise signal to noise. Finally, voxel-wise estimates of fractional anisotropy were derived using ‘dtifit’.

*Optic radiation tractography*

Probabilistic tractography was performed using FSL BedpostX [4]. For each hemisphere independently, connectivity distributions were computed between a 5mm sphere centred over the lateral geniculate nucleus (LGN) and a 10x15x10mm region of interest (ROI) mask placed to cover the upper and lower banks of the calcarine fissure, similar to approaches described previously [5,6]. ROIs for tractography were defined on the pre-operative anatomical scan for each patient, except the VFD patient lacking baseline imaging data (Case A) who was therefore not included in this analysis. An ‘exclusion’ mask was created to prevent tractography samples from a) extending through the splenium of the corpus callosum into the contralateral hemisphere, and b) crossing through the tail of the hippocampus. Probabilistic tractography was run using the default settings to generate 5000 samples. The resulting connectivity distribution maps were thresholded to exclude the 10% least probable streamlines, and binarised to create masks representing the left and right optic radiations.

*Tract based spatial statistics*

[7]. Between-group comparisons of FA were performed using the conventional Tract Based Spatial Statistics (TBSS) processing pipeline [7] . All participants’ FA images were nonlinearly aligned into a common space. Next, the mean FA image was created and thinned to form a mean FA ‘skeleton’, which represents the centres of all white matter tracts common to the group (Fig. 2a). In order to constrain analysis to the optic radiations, the Jülich histological atlas masks of the left and right optic radiations, thresholded at 10%, were applied to the skeleton. Each participant’s aligned FA data were then projected onto this skeleton, which was submitted to voxel-wise cross-subject permutation testing, implemented within FSL-‘randomise’ [8]. In order to examine FA changes ipsilateral and contralateral to the side of seizure onset / SLAH, the hemispheres of patients with right TLE were flipped to standardise the treatment hemisphere to the left across all patients.

*Choroidal fissure CSF volumes*

Cerebrospinal fluid (CSF) has been proposed to act as a cooling barrier around the MTL structures [1]. A previous case report noted narrowness of the choroidal fissure that surrounds the hippocampus in a patient who developed a VFD following SLAH [9]. Low CSF volumes could theoretically increase the risk of indirect heat injury to the LGN or white matter containing the optic radiations, even if these are not directly transected by the laser catheter [9,10]. To quantify CSF volume in the choroidal fissure, we created a ROI on the standard template MNI 2mm brain, using anatomical landmarks to define the temporal part of the choroidal fissure [11]. The inferior and lateral point was defined as the voxel medial to the lateral geniculate body based on the Harvard-Oxford atlas. Because we do not ablate posterior to the level of the tectal plate, the supero-posterior boundary of the choroidal fissure region of interest was placed at the posterior-most slice on which the tectal plate was visible. This standard ROI was nonlinearly aligned to each individual’s anatomical scan in order to manually select CSF voxels within this mask based on T1 intensity (Figure 3). The number of resulting voxels was converted into CSF volume (mm^3^), adjusted for intracranial volume using a subject-specific scaling factor calculated using the FSL tool Sienax [12].

*LGN tissue microstructure measurements*

We used the Jülich probabilistic histology atlas in order to define objective LGN masks for each hemisphere separately (Fig. 4). The Jülich probabilistic histology atlas LGN labels were thresholded to retain only those voxels histologically labelled as LGN in at least 33% of the atlas population. The resulting masks were visually inspected and corrected to exclude any voxels falling in CSF, the hippocampus or the brainstem. The atlas masks were nonlinearly aligned to each participant’s diffusion data to extract average FA, radial diffusivity (RD, thought to reflect myelination) and axial diffusivity (AD).

*SLAH parameters*

It has been proposed that heat exposure related to the amount of laser energy deposited may contribute to visual field deficits [10]. A single SLAH session typically involved between three and four treatment cycles, starting with a first ablation anteriorly in the amygdala followed by additional ablations targeting the anterior, middle and / or posterior hippocampus. The SLAH ablation parameters were available for 2 of 3 VFD patients and 8 of 10 SC patients. For each available dataset, we calculated the energy (Joules) deposited to the treated locations by multiplying the power (Watts) and the duration (seconds) of each ablation. The individual treatment cycles were also summed to obtain total energy deposited for every patient. Visual inspection indicated that ablations were limited to the amygdala, head and body of the hippocampus in the 2 VFD patients with available data. Consequently, we compared these 3 regions, as well as the total energy deposited (sum across all regions), between the VFD patients and SC. Volumes of the ipsilateral amygdala and hippocampus were included as covariates. These volumes were calculated using FSL-FIRST, manually corrected where necessary, and adjusted for head size as before.

**Supplementary Table S1. Optic radiations injury load.**

|  | Volume intersected (mm^3^) | Percent intersected (%) | Mean post-SLAH ipsilateral FA | Minimum post-SLAH ipsilateral FA (mean – 2SD) |
| --- | --- | --- | --- | --- |
| HC | - | - | 0.57 | 0.21 |
| SC | 6.1 ± 8.0 | 0.4 ± 0.5 | 0.52 | 0.13 |
| Case A | N/A | N/A | 0.53 | 0.23 |
| Case B | 0 | 0 | 0.56 | 0.17 |
| Case C | **32*** | 2 | 0.51 | **0.13*** |

*Legend*. Optic radiation lesion load measured using two complementary approaches. The trajectory of the laser catheter was masked on intraoperative MRI (available for 9 / 10 surgical controls, SC) to calculate spatial overlap with the ipsilateral optic radiations, reconstructed from the pre-SLAH diffusion tractography. Injury load was quantified both in terms of volume of intersection (mm^3^) and percent of optic radiation voxels affected by the catheter in the post-ablation SC group and in individual patients reporting visual field deficits (VFD) following ablation. No pre-operative diffusion data were available in Case A. Substantially greater lesion load was observed in one VFD patient (bold font) relative to the range (mean ± 2 standard deviations) in SC. To quantify changes to the diffusion parameters within the optic radiations, Fractional Anisotropy (FA) was compared between the visit 2 diffusion data for all groups (post-SLAH in patients). The ipsilateral optic radiations were standardised to be the left hemisphere in healthy controls (HC, n=15), surgical controls (SC, n= 10) and 3 patients with VFDs following ablation. Mean fractional anisotropy (FA) represents the average FA across all voxels in the optic radiation skeleton mask. Minimum FA was calculated separately for every voxel by measuring the value observed 2 standard deviations below the mean at every voxel location. Case A did not have baseline diffusion data. While the visual field deficits patient did not differ from control groups at baseline, following laser ablation Case C (bold font) showed ipsilateral minimum FA values lower than observed in both control groups (see Figure 2 for the location of these voxels).

**Supplementary Table S2. Baseline choroidal fissure CSF volumes and SLAH power**

| Choroidal fissure CSF volumes (mm^3^) | | | | |
| --- | --- | --- | --- | --- |
|  | Ipsilateral | | Contralateral | |
| HC | 620.5 ± 160.5 | | 700.5 ± 123.8 | |
| SC | 613.6 ± 126.9 | | 690.9 ± 114.1 | |
| Case A | - | | - | |
| Case B | 517 | | 630 | |
| Case C | 697 | | 749 | |
| SLAH energy deposited (Joules) | | | | |
|  | Amygdala | Anterior hippocampus | Mid hippocampus | Total |
| SC | 4885 ± 2812 | 4989 ± 2735 | 4071 ± 3051 | 16715 ± 9600 |
| Case A | - | - | - | - |
| Case B | 2084 | 3758 | 2144 | 7985 |
| Case C | 1989 | 2045 | 2540 | 6573 |

*Legend*. Cerebrospinal fluid (CSF) volumes measured within the choroidal fissure, adjusted for intracranial volume and laser energy deposited to the medial temporal lobe during selective laser amygdalohippocampotomy (SLAH). CSF volumes were measured from the baseline (visit 1) high resolution T1 weighted scans in healthy controls (n=15), surgical controls (n=10) and patients who would go on to report visual field deficits following laser ablation. Case A did not have a baseline scan available. In healthy controls, the left hemisphere was designated as ‘ipsilateral’. The amount of energy deposited by the laser during individual treatment cycles targeting sub-regions of the medial temporal lobe was determined by multiplying the power (Watts) and duration (in seconds) of each ablation in 2 VFD patients and 8 SC patients with available data.

**Supplementary Figure S1. Humphrey visual field assessment results in two patients who experienced a visual field deficit following SLAH.**

*
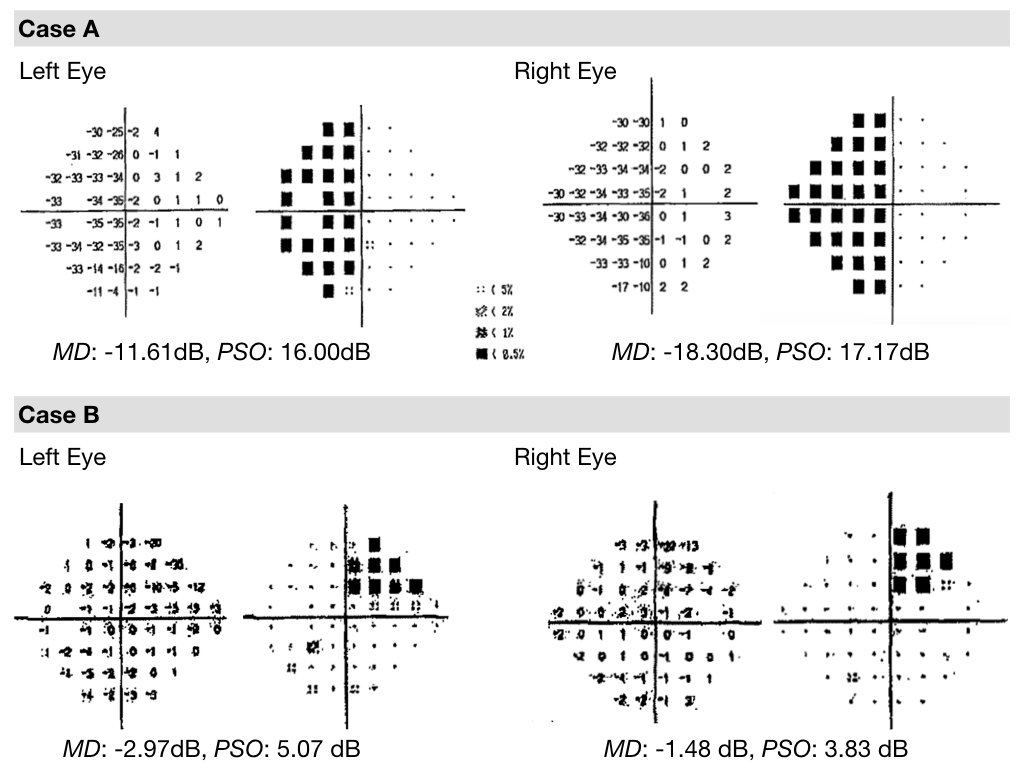
*

*Legend*. Ophthalmological test results (Humphrey Visual fields obtained using the 24-2 SITA – Fast protocol, total deviation plots) available for two patients who reported visual field symptoms following selective laser amygdalohippocampotomy (SLAH). Case A experienced a persistent (unchanged over 1 year) left homonymous hemianopsia following a repeat SLAH procedure to ablate the right hippocampus. Case B experienced a right superior quadrantanopsia after a left SLAH procedure that was noted to be barely noticeable by 6 months post-SLAH. Case C did not undergo a formal visual field examination. Test results are shown at 6 months post-SLAH for Case A and at 18 months post-SLAH for Case B. MD = mean defect. PSD = Pattern standard deviation.

**Supplementary Figure S2. Proximity of the laser catheter trajectory to the lateral geniculate nucleus in a visual field deficit patient.**

*
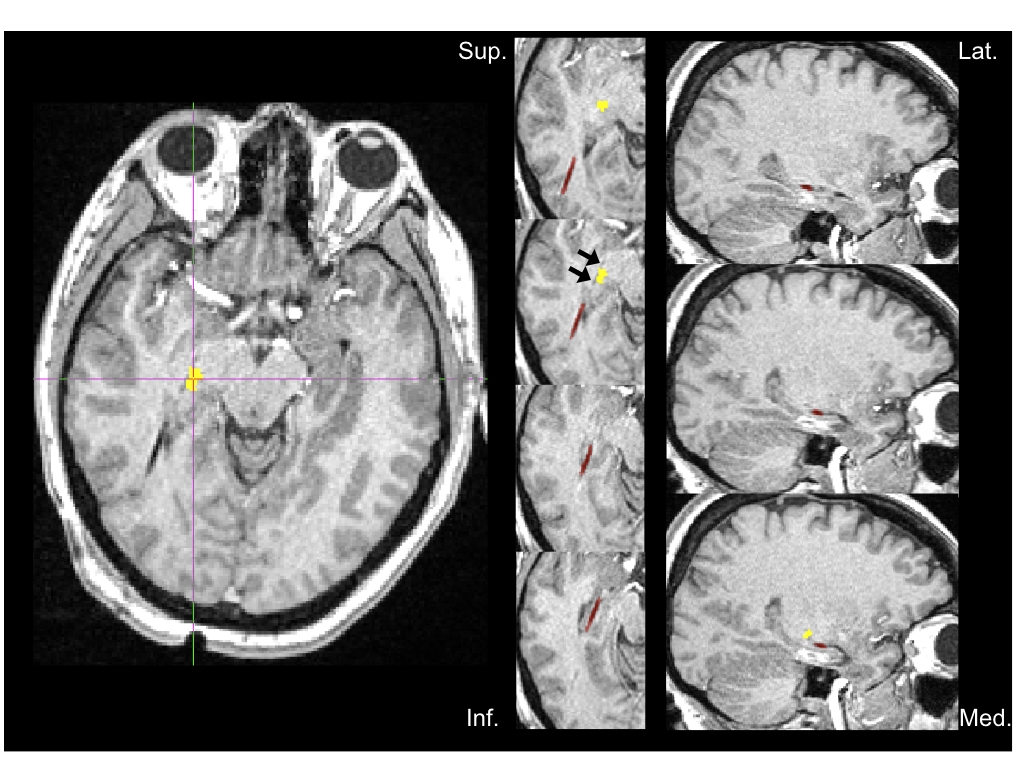
*

*Legend*. In Case A, the initial trajectory of the laser catheter passed close to the expected location of the LGN. Since the LGN cannot be precisely identified on T1-weighted anatomical scans, in order to visualise the relative course of the laser catheter (opaque red voxels), we overlaid the probabilistic location of the LGN from the Juelich post-mortem histological atlas (yellow) onto the patient’s intraoperative structural scan. While the main catheter trajectory coursed inferior to the LGN, artefact associated with the catheter’s placement was visible proximal to the infero-lateral border of the LGN. Sup = superior. Lat = lateral. Inf = inferior. Med = medial.

**Supplemental Figure S3. Laser energy delivered to medial temporal structures in patients with and without visual field defect.**

*
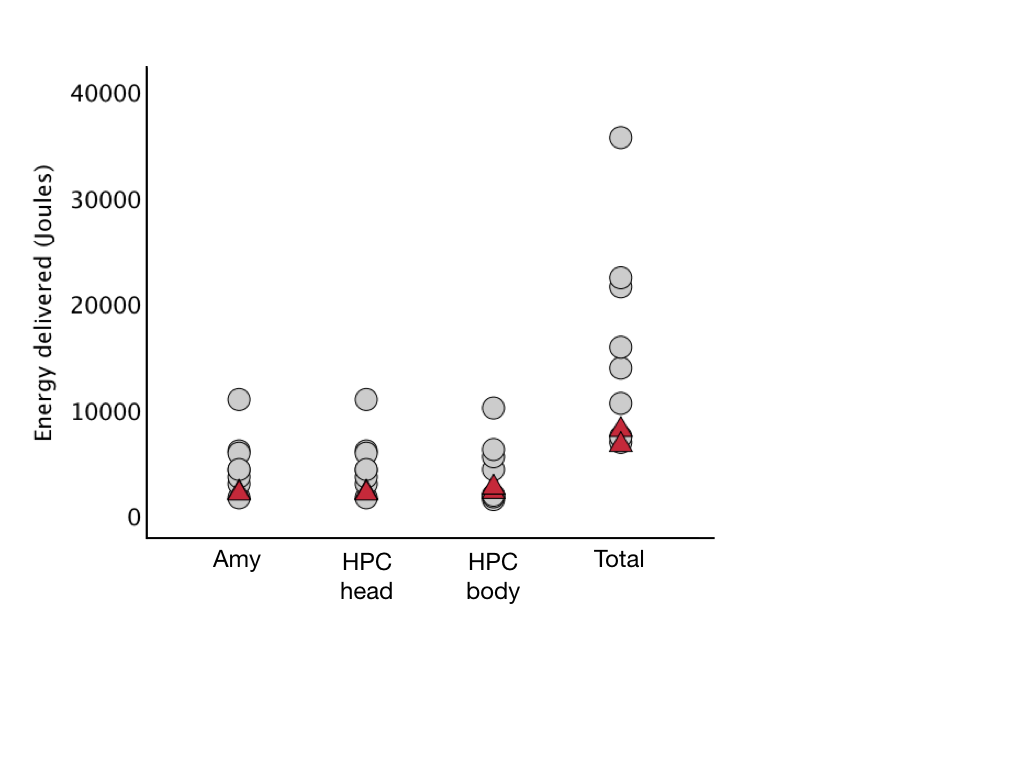
*

*Legend*. Regional and total laser energy deposited to medial temporal lobe structures during SLAH. Data were available for 2 of the patients who subsequently developed a visual field deficit (red triangles) and 9 of the 10 surgical controls (grey circles). No statistically significant difference was found. Amy = amygdala. HPC = hippocampus.

**Supplementary references**

1 Willie JT, Laxpati NG, Drane DL, Gowda A, Appin C, Hao C, Brat DJ, Helmers SL, Saindane A, Nour SG, Gross RE: Real-time magnetic resonance-guided stereotactic laser amygdalohippocampotomy for mesial temporal lobe epilepsy. Neurosurgery 2014;74:569-584; discussion 584-565.

2 Andersson JL, Skare S, Ashburner J: How to correct susceptibility distortions in spin-echo echo-planar images: application to diffusion tensor imaging. Neuroimage 2003;20:870-888.

3 Andersson JLR, Sotiropoulos SN: An integrated approach to correction for off-resonance effects and subject movement in diffusion MR imaging. Neuroimage 2016;125:1063-1078.

4 Behrens TE, Berg HJ, Jbabdi S, Rushworth MF, Woolrich MW: Probabilistic diffusion tractography with multiple fibre orientations: What can we gain? Neuroimage 2007;34:144-155.

5 Sherbondy AJ, Dougherty RF, Napel S, Wandell BA: Identifying the human optic radiation using diffusion imaging and fiber tractography. Journal of vision 2008;8:12 11-11.

6 Hofer S, Karaus A, Frahm J: Reconstruction and dissection of the entire human visual pathway using diffusion tensor MRI. Frontiers in neuroanatomy 2010;4:15.

7 Smith SM, Jenkinson M, Johansen-Berg H, Rueckert D, Nichols TE, Mackay CE, Watkins KE, Ciccarelli O, Cader MZ, Matthews PM, Behrens TE: Tract-based spatial statistics: voxelwise analysis of multi-subject diffusion data. Neuroimage 2006;31:1487-1505.

8 Winkler AM, Ridgway GR, Webster MA, Smith SM, Nichols TE: Permutation inference for the general linear model. Neuroimage 2014;92:381-397.

9 Jermakowicz WJ, Ivan ME, Cajigas I, Ribot R, Jusue-Torres I, Desai MB, Ruiz A, D'Haese PF, Kanner AM, Jagid JR: Visual Deficit From Laser Interstitial Thermal Therapy for Temporal Lobe Epilepsy: Anatomical Considerations. Oper Neurosurg (Hagerstown) 2017;13:627-633.

10 Waseem H, Vivas AC, Vale FL: MRI-guided laser interstitial thermal therapy for treatment of medically refractory non-lesional mesial temporal lobe epilepsy: Outcomes, complications, and current limitations: A review. Journal of clinical neuroscience : official journal of the Neurosurgical Society of Australasia 2017;38:1-7.

11 Nagata S, Rhoton AL, Jr., Barry M: Microsurgical anatomy of the choroidal fissure. Surgical neurology 1988;30:3-59.

12 Smith SM, Zhang Y, Jenkinson M, Chen J, Matthews PM, Federico A, De Stefano N: Accurate, robust, and automated longitudinal and cross-sectional brain change analysis. Neuroimage 2002;17:479-489.
